# Exhaled breath condensate as a potential specimen for diagnosing COVID-19

**DOI:** 10.4155/bio-2020-0083

**Published:** 2020-04-15

**Authors:** Maryam Khoubnasabjafari, Vahid Jouyban-Gharamaleki, Reza Ghanbari, Abolghasem Jouyban

**Affiliations:** ^1^Tuberculosis & Lung Diseases Research Center, Tabriz University of Medical Sciences, Tabriz, Iran; ^2^Pharmaceutical Analysis Research Center & Faculty of Pharmacy, Tabriz University of Medical Sciences, Tabriz, Iran; ^3^Digestive Oncology Research Center, Digestive Diseases Research Institute, Tehran University of Medical Sciences, Tehran, Iran

**Keywords:** clinical specimen, COVID-19, exhaled breath condensate

Coronavirus disease 2019 (COVID-19) is an emerging condition threatening the biosecurity of all nations on the planet. This pandemic of respiratory disease, caused by a novel β-coronavirus (called severe acute respiratory syndrome coronavirus 2 [SARS-CoV-2]), has high sequence similarity to SARA-CoV, which was responsible for a major outbreak in 2002–2003 [1]. Early diagnosis of suspected cases is a vital task to manage patients and to control the spread of the pathogen, especially as there can be a long period before the clinical symptoms of the infection present [2]. The current COVID-19 pandemic affects a wide range of parameters from the global economy to the personal behavior of human beings. In addition to important concerns to control the spread of the disease, further attention should be paid to the mental healthcare of members of society [3]. At the present time, the laboratory tests to diagnosis SARS-CoV-2 samples take between a number of hours and a number of days to complete, depending on the nature of the test [4]. Employing novel technologies, such as microfluidics, could provide a fast and accurate test for outbreaks [5].

The SARS-CoV-2 virus can be detected in variety specimens such as bronchoalveolar lavage fluid (BLF) [6], sputum [7], saliva [8] or by using throat [9] or nasopharyngeal swabs [8]. Reliable and rapid laboratory diagnosis of a SARS-CoV-2 infection is critical and experiments have revealed that nucleic acid detection by real-time reverse-transcription polymerase chain reaction (RT-PCR) is the clinical standard for diagnosis of patients with COVID-19 [4]. Replication of the specific regions of SARS-CoV-2 genome including coding area of virus S, E or N structural proteins or RNA-dependent RNA polymerase (RdRp) nonstructural protein allowed the development of RT-PCR tests for diagnosis of this novel virus in patients with COVID-19 [10]. Sequencing of the viral RdRp gene is also used for diagnosis, especially when the PCR result is not convincing for one target gene but the clinical/epidemiological suspicion for COVID-19 is high. In addition, whole genome sequencing of the virus genome RNA from positive samples provides more evidence for better understanding the genomic similarities between patients (molecular epidemiology) and possible mutations of the virus genome [1].

A retrospective analysis of different respiratory specimens (including nasal and pharyngeal swabs, BLF and sputum) was carried out to determine the reliability of the test at detecting SARS-CoV-2 in patients with COVID-19 infections [11]. Liu *et al.* concluded that nucleic acid test (NAT) is a rapid, easy to conduct and widely used laboratory diagnostic test for identifying the RNA of SARS-CoV-2. According to their findings, BLF exhibited the highest (100% cases) true positive results where nasal and pharyngeal swabs showed low (61% cases) true positive results for targeting the *ORF1ab* gene of the virus [11]. This is of note because the false negative results could provide misleading information for control and management of COVID-19. In another study, some failures in detecting SARS-CoV-2 in a proportion of samples apparently taken using an inappropriate technique were reported [12]. Furthermore, in a recent study [13], biodistribution of SARS-CoV-2 was evaluated in 205 COVID-19 patients using real time RT-PCR targeting *ORF1ab*. The researchers collected different clinical specimens from patients across multiple sites and demonstrated that the type of sample collected can affect the result of virus diagnosis as well as influence accurate identification of patients with COVID-19. According to their results, the BLF specimen showed the highest rate of true positive results (14 of 15; 93%) to identify patients, with the lowest false negative results. However, it should be considered that this cannot be recommended for all cases in the screening stage as this is an invasive sampling procedure and requires highly skilled professionals to collect the BLF samples. BLF is collected by a bronchoscope passed through the mouth or nose into the lung, then a given volume of a sterile solution (usually NaCl 0.9%) is introduced to the lung and the fluid is collected for further biochemical examinations [14].

Since the virus spreads via respiratory droplets, and the success rate of BLF is better than sputum, nasal and pharyngeal swabs [6,11,13], exhaled breath condensate (EBC) could be considered a more appropriate sample to follow virus NAT using RT-PCR due to its similarities with BLF (i.e., biochemical contents and origin of production). EBC is a condensed form of small droplets of lung lining fluid [15] which is normally exhaled and contains a variety of components from small ions to proteins and organelles, even viruses, fungi and bacteria [16–18]. In a recent article [19], some technical tips for improving the quality and quantity of extracting NAT from EBC samples were reported. The same procedure with some modifications could be used to detect the genome of SARS-CoV-2 by using RT-PCR. EBC samples could be easily collected using a simple cold trap, commercially available EBC sampling device (such as EcoScreen^®^ or RTube^®^) or even using a tube passing water–ice mixture. The mechanism of sample collection by these devices is cooling down the temperature of the collection chamber from 0 to -25°C as has been reviewed in recent works [15,20]. Ahmadzai *et al.* [21] compared the efficacy of different collection devices for measuring the variations of biomarkers in EBC samples. Despite BLF, collection of EBC is simple, well tolerated by sample donors and no adverse effects have been reported so far, therefore it could be employed for sampling on a large scale to screen the suspected patients in viral epidemics such as the recent pandemic.

## Conclusion

We propose that EBC samples should be tested as a noninvasive sampling method in clinics, since it seems a promising specimen for diagnosis of patients with COVID-19 infections. EBC sampling could be performed as many times as needed in follow-up investigations of patients, which is not possible with BLF sampling. Unfortunately, our team does not currently have access to a safety level 3 laboratory in our research center to test the applicability of this hypothesis. There is also a possibility of developing microfluidics or other single-use technologies to collect and analyze the samples to provide faster screening tools in pandemics such as COVID-19.
